# Social Support and Self-Reported Stress Levels in a Predominantly African American Sample of Women with Systemic Lupus Erythematosus

**DOI:** 10.1155/2015/401620

**Published:** 2015-09-09

**Authors:** Edith Marie Williams, Jiajia Zhang, Judith Anderson, Larisa Bruner, Laurene Tumiel-Berhalter

**Affiliations:** ^1^Department of Public Health Sciences, Medical University of South Carolina, 135 Cannon Street, Suite 303, Charleston, SC 29425, USA; ^2^University of South Carolina, Columbia, SC 29208, USA; ^3^State University of New York at Buffalo, Buffalo, NY 14215, USA

## Abstract

Lupus patients should avoid stress because physical or emotional stress can affect overall physical health. It has been suggested that social support has a positive influence on health status, but there is a lack of information in the literature on the association between the two among lupus patients. The current study investigated the association between social support and self-reported stress and coping status among African American women with lupus using data collected from two linked cross-sectional surveys. No social support differences in groups of high and low stress/coping were revealed; a duplicate study with a larger sample size is required.

## 1. Introduction

Stress can be a physical, mental, and/or emotional response to life's changes and demands. Stress can have immediate and chronic effects. The stress response is the set of physical and emotional changes the human body makes in response to a threat or stress [1]. Stress has been linked to chronic disease because if it occurs too often or lasts too long, the immune system can be weakened, making it harder to fight off disease and/or worsening existent health problems. Thus, stress is a particularly important issue for lupus patients, since lupus is an autoimmune disease wherein the immune system is already dysfunctionally attacking the body's own cells. Lupus patients are advised to avoid stress and to try to lead lives that are close to normal because physical or emotional stress can trigger an exacerbation and affect overall physical health [2–4]. It has been suggested that social support or the extent to which resources in the social environment meet an individual's interpersonal needs has a positive influence on health status. Social support has gained much research attention due to its believed beneficial effects on the psychosocial and physical well-being of lupus patients [5–10].

Some researchers have examined both stress and social support and their effects on the mental and physical health of SLE patients, but not their relation to each other. Dobkin and associates [11] assessed daily hassles, social support, psychologic distress, and quality of life in 44 women with SLE, using standardized instruments, and found that high stress (assessed by hassles severity) and poor social support were the strongest predictors of patients' perceived global physical health and psychologic distress. Other variables investigated included SLE disease activity and damage, age, disease duration, education, and ethnicity [11].

In addition to life stress and social support, Kozora et al. [12] examined coping styles in relation to psychological processes in SLE and Rheumatoid Arthritis (RA) patients. Investigators measured depression, mood, disease activity, perceived health, and cognitive ability and found that increased depressive symptoms and distressed mood state in SLE patients were related to use of disengaging and emotional coping styles [12]. Dobkin and colleagues [13] assessed psychological distress, social support, stress, coping, and health-related quality of life. They observed that better physical health was predicted by more emotion-oriented coping in patients in a more active disease state, suggesting that this style of coping may be more adaptive in situations that are considered uncontrollable (e.g., SLE flare) [13]. McCracken and associates [14] investigated the relations of coping with psychological adjustment and functional status in 46 adults with SLE and found that seeking social support was a significant predictor of adjustment, although neither perceived nor tangible social support was measured in relation to coping [14].

While the effects of stress and social support on disease activity in lupus patients have been well-documented, there is lack of information in the literature on the association between social support and stress levels among lupus patients. Therefore, the objective of this study was to determine if there is a link between social support and self-reported stress and coping status among African American women with lupus.

## 2. Patients and Methods

In brief, this paper reflects the analysis stress/coping and social support data, collected from two linked cross-sectional surveys. Cases analyzed for the current study were participants in the Buffalo Lupus Project and Breakfast with a Buddy Biomarkers of Lupus Study. The Buffalo Lupus Project, a partnership between the Toxic Waste Lupus Coalition and the University at Buffalo, was formed to count the number of people in the Buffalo community who had lupus and other autoimmune diseases and also to uncover any common factors that may elucidate the complex causes of lupus and other autoimmune diseases. At its onset, the Buffalo Lupus Project consisted of a registry and survey. The survey component of the Buffalo Lupus Project focused on the east side of the city of Buffalo, NY, which is predominantly African American. These areas were targeted due to citizens' concern about high rates of autoimmune disease in the area that they believed may be related to the presence of a toxic waste site there. Trained interviewers completed surveys with 66 patients with SLE, 52 of whom were African American women. Survey topics included demographics, diagnosis, health care utilization, residential history, residential history exposure, occupational history, occupational history exposure, smoking history, family health history, and social support.

Upon completion of the survey, each participant was invited to participate in the Breakfast with a Buddy Biomarkers of Lupus Study, which was developed to investigate the relationship between inflammation and heart disease in African American women with lupus. Participation in the study included a fasting blood draw, carotid ultrasound, physical measurements, and a short questionnaire assessing traditional cardiovascular risk factors, including stress/coping status.

Currently, 317 residents with lupus and other autoimmune diseases have registered with the Buffalo Lupus Project. Out of the 317 current registrants, 194 have reported being diagnosed with SLE, and, of those SLE cases, 92 were eligible to participate in the Buffalo Lupus Project survey due to having ever lived or currently living in zip codes 14211 or 14215. Sixty-six SLE cases completed the survey and all were invited to participate in the biomarkers study; 44 consented, corresponding to a 67 percent response rate. Responses to the social support variables from the Buffalo Lupus Project survey were merged with the Breakfast with a Buddy Biomarkers of Lupus Study database, and those cases who had participated in both projects and responded to both social support and stress questions were selected out for analysis. Forty-three cases had available values for both stress and social support inquiries. After exclusion of those cases with “Do not know/Not applicable” (DK/NA) responses for the dichotomized stress/coping variable, 41 cases were included in analysis.

Stress and coping status was measured as self-reported perceived level of stress and ability to cope, using a question, adapted from the American Heart Association (AHA) Coronary Risk Assessment Questionnaire [15], which asked the participants to describe how they currently coped with stress in their life using a five-item scale (seldom feeling stress, coping very well; sometimes stressed, coping fairly well; often stressed, having trouble coping at times; heavily stressed, often having trouble coping; or excessively stressed, or unable to cope). Responses were dichotomized for analysis. Specifically, “seldom” and “sometimes” responses were coded as no/low stress, and “often,” “heavily,” and “excessively” responses were codes as yes/high stress. Social support was assessed by six questions, adapted from the Social Support Questionnaire 6 and Medical Outcomes Study Short Form Health Survey (SF-36) [6, 16–21]. Five themes were evaluated: perception of having a support system and individuals/groups comprising it, proximity and number of perceived close friends and family members, perception of having someone there to take over tasks and chores if sick, membership and attendance at faith based and other types of meetings, and frequency of get-togethers and conversations with friends, relatives, and neighbors.

Participants were asked how many close friends or relatives they saw at least one time per month (none through 5 or more), how many times in a typical week participants talk on the phone (more than once per week, once per week, and less than once per week), and how often patients get together with friends or relatives (more than once per week, less than once per month, once per week, never or almost never, and 1–3 times per month). Participants were also asked if they had a support system, if they lived with someone they considered to be a close friend or relative, and if they belonged to any clubs and/or organizations. Confounding variables assessed included age, highest level of education attained, annual household income, smoking status, and race.

The stress/coping variable was cross-tabulated with each of the six social support variables investigated, using SPSS, and percentages were calculated. Statistical tests were conducted based on the Fisher exact test and *t*-test due to the small sample size by assuming the independence among the high and low stress group.

## 3. Results

Approximately 88 percent of participants were African American and the remaining 12 percent were Caucasian. The mean age of participants was 50.8 years. Less than ten percent of participants completed less than 12 years of school. Approximately 77 percent of participants graduated from high school, and 17 percent attained a college degree or higher.  Table 1 shows that 28 participants were classified as “no/low stress.” The remaining 13 participants were classified as “yes/high stress.” When divided into categories of low and high stress, those reporting higher levels of stress were slightly younger than those in the “no/low stress” category (mean age of 49 years compared with 52 years). There were more White participants in the “yes/high stress category” than there were in the “no/low stress category.” Conversely, there were more Black participants in the “no/low stress category” than there were in the “yes/high stress category.” In both categories, the majority of participants reported attending some college or attaining a college degree or higher. In the “yes/high stress category,” the majority of respondents reported annual household incomes below $30,000.00. Conversely, in the “no/low stress category,” the most respondents reported annual household incomes at or above $30,000.00. In both stress categories, few participants reported being current smokers (less than 16 percent).

Table 2 shows that most of the SLE patients in the current study (97.6 percent) had a support system that was comprised of both immediate family members and unrelated sources of support. While the majority of participants in both stress categories reported their family, children, siblings, other family members, and friends to be part of their support systems, more participants in the “yes/high stress” category reported their spouse and a spiritual leader to be part of their support system compared to participants classified as “no/low stress” (*P* = 0.02). Additionally, approximately half of participants in the “no/low stress” category reported coworkers to be a part of their support system, while less than a quarter of participants in the “yes/high stress” category (23.1 percent) reported coworkers to be a part of their support system. More than half of participants reported living with someone they considered a close friend or relative, and the majority of participants reported belonging to at least one club or organization (73 percent).

Few differences were observed between the two stress categories with respect to the six social support variables investigated (Table 3). In both categories, the majority of participants reported having four or more close friends and/or relatives they see at least once per month (68 percent of no/low stress group and 69 percent of yes/high stress group) and talking on the phone with family, friends, and/or neighbors more than once per week (86 percent of no/low stress group and 92 percent of yes/high stress group). The largest number of participants in both of the stress/coping categories reported getting together with friends and/or relatives one to three times per month or once per week or more. However, a smaller percentage of participants in the “no/low stress” category reported getting together with friends and/or relatives one to three times per month when compared with participants in the “yes/high stress” category (32 percent of the no/low stress group and 54 percent of the yes/high stress group), and a smaller percentage of participants in the “yes/high stress” category reported getting together with friends and/or relatives once per week or more when compared with participants in the “no/low stress” category (31 percent of the yes/high stress group and 50 percent of the no/low stress group). In both categories, the majority of participants perceived themselves to have a support system (96 percent of no/low stress group and 100 percent of yes/high stress group) and live with someone they consider a close friend and/or relative (61 percent of no/low stress group and 62 percent of yes/high stress group). The same trend was observed with respect to club/organization membership. In both categories, the majority of participants reported belonging to a club or organization, although a smaller percentage of participants in the “no/low stress” category reported this trend when compared with participants in the “yes/high stress” category (68 percent of no/low stress group and 85 percent of yes/high stress group).

## 4. Discussion

The current study did not observe social support differences in groups of high and low stress/coping from a predominantly African American sample of middle-aged women with SLE. A key finding indicates that most SLE patients in the current study had social support.

There are a variety of reasons where a link between higher levels of social support and lower levels of stress was not observed. There may, in fact, not be a relationship between the two factors. However, statistical tests were not conducted due to small overall sample size and inability to compare such small groups as independent samples when divided into groups of high and low stress. It will require a duplicate study with a larger sample size to determine if findings are reproducible and statistically valid. It is more likely that the homogeneity of the study sample made it impossible to observe existent trends. In addition to very little variability with regard to social support, there was very little variability in the sample with regard to race and education. However, the slight differences in age, race, and/or income (proxy marker of SES) between the two stress categories could have masked the effects of social support. It is also possible that the way social support and stress/coping were assessed was not comprehensive enough and, therefore, did not truly measure what the present study aimed to measure. Other studies of stress and coping in SLE populations used entire scales, such as the Daily Stress Inventory and Daily Hassles Questionnaire [4, 22–26], whereas the current study assessed stress and coping using one question, although the question incorporates response choices reminiscent of such validated scales. The scales used may not have been appropriate since participants usually want to report what they think the interviewer wants to hear. Misclassification may have occurred due to such response bias. Since other studies have not examined stress and social support in relation to one another, present results could not be compared to those of other investigators. It was assumed, however, that a positive relationship between stress and social support would be observed, similar to the positive relationships between social support and physical well-being of lupus patients observed by other researchers [5–10].

It cannot be ignored, however, that social support is one of the most important factors in predicting the physical health and well-being of everyone, ranging from childhood to older adults. The absence of social support shows some disadvantage among the impacted individuals. In most cases, it can predict the deterioration of physical and mental health among the victims. The initial social support given is also a determining factor in successfully overcoming life stress. Studies in the general population have observed that the presence of social support significantly predicts the individual's ability to cope with stress. Knowing that they are valued by others is an important psychological factor in helping them to forget the negative aspects of their lives and think more positively about their environment. Not only does social support help improve a person's well-being, it affects the immune system as well. Thus, it is also a major factor in preventing negative symptoms such as depression and anxiety from developing [27]. While the present study did not observe differences in an SLE cohort, broken into groups of high and low stress with respect to amount of social support perceived, it is an area that should be explored further. Health status and disease activity in patients with SLE and other chronic diseases are strongly associated with social support, which suggests that enhanced social support in medical care might improve SLE outcome.

## Figures and Tables

**Table 1:** Demographic characteristics of SLE cases in the Buffalo Lupus Project and Breakfast with a Buddy Biomarkers of Lupus Study (*N* = 41).

	Total (41)	Low stress (28)	High stress (13)	*P* value
Age, years, mean (SD)		52 (13.11)	49 (5.48)	0.3084
Education, *n* (%)				0.3523
<Grade 12	2 (4.9)	2 (7.1)	0 (0)	
High school grad	11 (26.8)	7 (25.0)	4 (30.8)	
Some college	19 (46.3)	11 (39.3)	8 (61.5)	
College graduate	9 (22.0)	8 (28.6)	1 (7.7)	
Annual household income, *n* (%)				0.5815
Under $10,000	7 (17.1)	4 (14.3)	3 (23.1)	
$10,000–29,999	15 (36.6)	9 (32.1)	6 (46.2)	
$30,000–49,999	8 (19.5)	7 (25.0)	1 (7.7)	
Over $50,000	8 (19.5)	5 (17.9)	3 (23.1)	
Smoking status, *n* (%)				0.6666
Never	17 (41.5)	13 (46.4)	4 (30.8)	
Past (quit)	18 (43.9)	11 (39.3)	7 (53.9)	
Current	6 (14.6)	4 (14.3)	2 (15.4)	
Race, *n* (%)				0.5698
African American	36 (87.8)	26 (92.9)	10 (76.9)	
White	5 (12.2)	2 (7.1)	3 (23.1)	

*N*: total number of participants, *n*: number of participants in each cell, %: percent, and SD: standard deviation.

**Table 2:** Social support characteristics of SLE cases in the Buffalo Lupus Project and Breakfast with a Buddy Biomarkers of Lupus Study (*N* = 41).

Social support parameters	Total (41)	Low stress (28)	High stress (13)	*P* value
Having support system, *n* (%)	40 (97.6)	27 (96.4)	13 (100)	1
Family	38 (92.7)	25 (89.3)	13 (100)	0.5390
Spouse	23 (56.1)	12 (42.9)	11 (84.6)	0.0181^*∗*^
Children	35 (85.4)	23 (82.1)	12 (92.3)	0.6448
Siblings	29 (70.7)	19 (67.9)	10 (76.9)	0.7186
Other family members	30 (73.2)	20 (71.4)	10 (76.9)	1
Coworkers	16 (39.0)	13 (46.4)	3 (23.1)	0.1874
Friends	35 (85.4)	23 (82.1)	12 (92.3)	0.6448
Spiritual leader	31 (75.6)	19 (67.9)	12 (92.3)	0.1286
Support group	9 (22.0)	5 (17.9)	4 (30.8)	0.4288
Living with someone considered close friend and/or relative, *n* (%)	25 (61.0)	17 (60.7)	8 (61.5)	1
Belonging to clubs/organizations, *n* (%)	30 (73.2)	19 (67.9)	11 (84.6)	0.4507

*N*: total number of participants, *n*: number of participants in each cell, %: percent, and SD: standard deviation.

*∗* indicates statistical significance.

**Table 3:** Social support characteristics of SLE cases in categories of high and low stress (*N* = 41).

Characteristics	Total (41)	Low stress (28)	High stress (13)	*P* value
Number of close friends/relatives seen at least once per month, *n* (%)				1
None	3 (7.3)	2 (7.1)	1 (7.7)	
1–3	10 (24.4)	7 (24.9)	3 (23.1)	
4 or more	28 (68.3)	19 (67.9)	9 (69.3)	
Number of times of talk on the phone with family/friends/neighbors in a typical week, *n* (%)				1
More than once	36 (87.8)	24 (85.7)	12 (92.3)	
Once per week	5 (12.2)	4 (14.3)	1 (7.7)	
How often patients get together with friends/relatives, *n* (%)				0.6453
Once per week or more	18 (43.9)	14 (50.0)	4 (30.8)	
1–3 times per month	16 (39.0)	9 (32.1)	7 (53.8)	
Less than once/month	3 (7.3)	2 (7.1)	1 (7.7)	
Never/almost never	3 (7.3)	2 (7.1)	1 (7.7)	
Having support system, *n* (%)				1
Yes	40 (97.6)	27 (96.4)	13 (100)	
No	1 (2.4)	1 (3.6)	0 (0)	
Living with close friend/relative, *n* (%)				1
Yes	25 (61.0)	17 (60.7)	8 (61.5)	
No	15 (36.6)	10 (35.7)	5 (38.5)	
Belonging to any clubs/organizations, *n* (%)				0.2307
Yes	30 (73.2)	19 (67.9)	11 (84.6)	
No	10 (24.4)	9 (32.1)	1 (7.7)	

*N*: total number of participants, *n*: number of participants in each cell, %: percent, and SD: standard deviation.
